# Biopolymer Hydrogel Scaffolds Containing Doxorubicin as A Localized Drug Delivery System for Inhibiting Lung Cancer Cell Proliferation

**DOI:** 10.3390/polym13203580

**Published:** 2021-10-17

**Authors:** Chuda Chittasupho, Jakrapong Angklomklew, Thanu Thongnopkoon, Wongwit Senavongse, Pensak Jantrawut, Warintorn Ruksiriwanich

**Affiliations:** 1 Department of Pharmaceutical Sciences, Faculty of Pharmacy, Chiang Mai University, Chiang Mai 50200, Thailand; pensak.amuamu@gmail.com (P.J.); warintorn.ruksiri@cmu.ac.th (W.R.); 2 Cluster of Research and Development of Pharmaceutical and Natural Products Innovation for Human or Animal, Chiang Mai University, Chiang Mai 50200, Thailand; 3 Department of Biomedical Engineering, Faculty of Engineering, Srinakharinwirot University, Nakhon Nayok 26120, Thailand; jakrapong.a@hotmail.com (J.A.); wongwit@g.swu.ac.th (W.S.); 4 Department of Pharmaceutical Technology, Faculty of Pharmacy, Srinakharinwirot University, Nakhon Nayok 26120, Thailand; thanu@g.swu.ac.th

**Keywords:** gelatin, sodium carboxymethyl cellulose, scaffold, A549 cells, freeze drying

## Abstract

A hydrogel scaffold is a localized drug delivery system that can maintain the therapeutic level of drug concentration at the tumor site. In this study, the biopolymer hydrogel scaffold encapsulating doxorubicin was fabricated from gelatin, sodium carboxymethyl cellulose, and gelatin/sodium carboxymethyl cellulose mixture using a lyophilization technique. The effects of a crosslinker on scaffold morphology and pore size were determined using scanning electron microscopy. The encapsulation efficiency and the release profile of doxorubicin from the hydrogel scaffolds were determined using UV-Vis spectrophotometry. The anti-proliferative effect of the scaffolds against the lung cancer cell line was investigated using an MTT assay. The results showed that scaffolds made from different types of natural polymer had different pore configurations and pore sizes. All scaffolds had high encapsulation efficiency and drug-controlled release profiles. The viability and proliferation of A549 cells, treated with gelatin, gelatin/SCMC, and SCMC scaffolds containing doxorubicin significantly decreased compared with control. These hydrogel scaffolds might provide a promising approach for developing a superior localized drug delivery system to kill lung cancer cells.

## 1. Introduction

Lung cancer is the leading cause of death in men and women, responsible for almost 25% of all cancer deaths [1]. Chemotherapy is the first-line therapy for small-cell lung cancer (SCLC) pre-operation and post-operation, and stage IV non-small cell lung cancer (NSCLC) [2]. Chemotherapy is also used as an adjuvant chemotherapy to kill remaining cancer cells and to reduce the chance of recurrence. In addition, adjuvant chemotherapy shrinks the tumor before surgery and reduces the spread of cancer, making surgery less invasive and more effective. Chemotherapy is commonly administered to patients intravenously at the maximum tolerable doses, which may cause severe toxicity in healthy tissues [3]. Although patient survival rates from chemotherapy treatments are high, drug resistance and drug-related toxicity severely limit clinical outcomes [4]. Doxorubicin (DOX) is an anthracycline type of chemotherapy, widely used for the treatment of solid tumors, including lung tumor [5]. A DOX HCl injection causes cumulative and dose-dependent cardiotoxicity. Cardiotoxicity, which develops at later stages of therapy or after the treatment, can cause severe cardiomyopathy and congestive heart failure, leading to patient death [6]. The administration of a DOX HCl injection does not efficiently deliver the drug to the target site at a therapeutic concentration and fails to maintain sufficient drug accumulation in the tumor. Some fractions of the drug administered to the patients reach tumor cells and some may transport to other tissues, leading to a reduced efficacy and an increased toxicity in normal tissues [7].

Scaffold, a localized drug delivery system, is a promising platform for maintaining the drug concentration in the blood circulation at low levels while increasing the drug level at the therapeutic concentration that reaches tumor cells. The scaffolds can release the drug to kill the remaining tumors that remain due to an incomplete surgical removal of the tumor cells from the lung, the main cause of recurrence and metastasis [8]. The localized drug delivery systems include a tumor proximity implant and an intra-tumoral injection. The retention of the chemotherapeutic drug at the tumor site decreases drug concentration in the blood circulation, hence limiting drug exposure to other normal tissues [9,10]. The drug retained at the tumor ensures the therapeutic efficacy of the drug. Hydrogel scaffolds have many advantages, including a high drug encapsulation efficiency, a controlled and sustained release of chemotherapeutic drugs, and a high water uptake capability with both solid and liquid properties [11]. The properties of the hydrogel scaffold such as morphology, pore size and the drug release profile can be modulated through a ratio of the polymer mixture [12,13]. 

Gelatin is a natural biomaterial with the advantages of biocompatibility, biodegradability, low immunogenicity, low cost, and includes many functional groups that allow for structure modification [14]. However, gelatin is sensitive to enzymatic degradation. SCMC is a chemically modified water-soluble polysaccharide widely used as a drug delivery system and drug delivery matrix. The advantages of SCMC are its high biocompatibility, biodegradability, and low immunogenicity [15]. Crosslinked SCMC has a high water absorption capacity and a suitable swelling degree to form hydrogel with a dynamic viscoelastic property [16]. In this study, the hydrogel scaffold encapsulating the DOX was constructed using a varying gelatin and SCMC ratio. The effects of a crosslinker on the scaffold morphology and pore size were observed. The water swelling capacity and drug release kinetic profile of the hydrogel scaffold loaded with DOX were characterized. The anti-proliferative effect of the scaffolds against the lung cancer cell line was investigated. We hypothesized that the addition of SCMC to gelatin may present a promising approach for developing a superior localized drug delivery hydrogel scaffold by improving morphology, pore size, drug loading efficiency, the swelling property, and drug release profile. 

## 2. Materials and Methods

### 2.1. Materials

Gelatin, sodium carboxymethyl cellulose (SCMC) (viscosity = 400–1000 mPa.s, degree of substitution = 0.60–0.95), and glutaraldehyde 25% aqueous solution (Sigma–Aldrich Inc., St. Louis, MO, USA). The DOX HCl injection solution (Pfizer Inc. New York, NY, USA). A549 cells, a human adenocarcinoma cell line derived from lung cancer cell of a male patient aged 58 (Japanese Collection of Research Bioresources (JRCB) Cell Bank, Tokyo, Japan). Dulbecco′s Modified Eagle Media (DMEM), fetal bovine serum (FBS), penicillin–streptomycin (10,000 U/mL of penicillin and 10,000 U/mL of streptomycin), and MTT (3-(4,5-dimethylthiazol-2-yl)-2,5-diphenyltetrazolium bromide) (Life Technologies Inc., Carlsbad, CA, USA).

### 2.2. Preparation of Gelatin-SCMC Porous Scaffold

The porous scaffolds, generated from a gelatin and SCMC mixture at different ratios, were fabricated using the swelling and freeze drying processes. Gelatin powder was dissolved in distilled water at 70 °C to obtain a 3% *w*/*v* gelatin solution. Sodium carboxymethyl cellulose (SCMC) was dissolved in distilled water at a concentration of 2% *w*/*v*. The two polymer solutions were mixed at different ratios, i.e., 10:0, 9:1, 8:2, 7:3, 6:4, and 5:5 under constant stirring. The polymer mixture was poured into 24-well plates. The glutaraldehyde solution (2% *w*/*v*) was added dropwise to provide the final concentration at 0.2% *w*/*v*. The resulting hydrogel was washed three times with glycine (0.1 M) and distilled water to inactivate unreacted aldehyde groups. The hydrogels were swollen at 4 °C for 24 h, and were then kept at −80 °C for 18 h. Then, the frozen hydrogel was freeze-dried for 48 h to form a porous scaffold. For the scaffold containing the anti-cancer drug, DOX (167 µg/mL) was added into the polymer mixture before crosslinking. The dried scaffolds were stored at 4 °C until use. 

### 2.3. Scaffold Morphological Investigation by Scanning Electron Microscopy

The morphology of the scaffolds was analyzed using the HITACHI S-3400N scanning electron microscope (Hitachi High-Tech in America Inc., Schaumburg, IL, USA) to evaluate their structure and porosity. Scaffold samples were fixed on an aluminum stub with conductive double-sided adhesive tape and coated with gold in an argon atmosphere prior to the photograph [17]. The images were captured at 100× magnification (20 kV). The average pore sizes for each scaffold were randomly measured for Martin’s diameter and were recorded to obtain an average with a standard deviation.

### 2.4. Scaffold Swelling Capacity Determination

The swelling capacity of scaffolds was tested by measuring their water uptake capacity. The scaffolds were immersed in a phosphate-buffered saline (PBS), pH 7.4. Portions of the scaffolds were cut and weighed, measuring at around 2.5 mg. The initial weight of the scaffold was recorded (*W*_0_). The scaffolds were then immersed in 20 mL of PBS, pre-incubated at 37 °C for 45 min and at 37 °C for 5 h. The scaffolds were removed, and the surface adsorbed PBS was removed using filter paper. The scaffolds were weighed again (*W*_1_). The swelling degree percentage of the porous scaffold was calculated using Equation (1), and the average value was obtained from the three experiments.

(1) $$Swelling\;degree\;\left(\%\right)=\frac{W_{1}-W_{0}}{W_{0}}\times 100\%$$

where *W*_0_ is the weight of the scaffold before swelling and *W*_1_ is the weight of scaffold after swelling.

### 2.5. Drug Encapsulation and Loading Efficiency in Scaffold

The scaffold containing DOX was cut and weighed, measuring at around 2.5 mg. The scaffolds were dissolved in deionized water (500 µL) under sonication until they were completely dissolved. The solution was analyzed to detect the amount of encapsulated the DOX using a fluorescence microplate reader (Spectramax M3, Molecular Devices Inc., San Jose, CA, USA) at Ex. 482 nm and Em. 590 nm [18]. A calibration curve was constructed by plotting the mean fluorescent intensity of DOX in deionized water versus concentrations ranging from 0.5–500 µg/mL. The linear regression method was used for drug quantitation. The DOX concentration in the scaffold samples was calculated according to a linear equation of a standard curve. The absorbance of the blank scaffolds was subtracted from that of the samples. The encapsulation efficiency (%) and loading efficiency (%) were calculated using Equations (2) and (3), respectively.

(2) $$Encapsulation\;efficiency\;\left(\%\right)=\frac{Amount\;of\;DOX\;in\;the\;scaffold\;}{Amount\;of\;DOX\;initially\;added}\times 100\%$$

(3) $$Loading\;efficiency\;\left(\%\right)=\frac{Amount\;of\;DOX\;in\;the\;scaffold\;}{Weight\;of\;Scaffold}\times 100\%$$

### 2.6. In Vitro Drug Release of Scaffold

The scaffolds containing doxorubicin were cut to provide a 2.5 mg mass, containing 16.7 µg of DOX. Each sample was immersed in 500 µL of PBS, pH 7.4 and incubated at 37 °C for 15, 30, 60 min and 24 h. At pre-determined intervals, samples were centrifuged at 13,000 rpm for 10 min. The amount of DOX released into the supernatant was measured using a fluorescence microplate reader at Ex. 482 and Em. 590 nm (Spectramax M3, Molecular Devices Inc., San Jose, CA, USA). A calibration curve was constructed by plotting mean fluorescent intensity of the DOX in PBS versus the DOX concentrations ranging from 0.5–500 µg/mL. The cumulative release of DOX (%) was calculated using Equation (4).

(4) $$Cumulative\;release\;of\;DOX\;\left(\%\right)=\frac{Amount\;of\;DOX\;released\;from\;the\;scaffold\;}{Amount\;of\;DOX\;loaded\;in\;the\;scaffold}\times 100\%$$

### 2.7. Cell Culture

The A549 cells were cultured in Dulbecco′s Modified Eagle′s Medium (DMEM), supplemented with 10% FBS and 1% penicillin–streptomycin, and maintained in a humidified incubator with an atmosphere of 5% CO_2_, at 37 °C (19).

### 2.8. In Vitro Cell Viability Study

The A549 cells (2.5 × 10^3^ cells/mL) were developed in a 24-well plate for 24 h. Both scaffolds containing DOX (42 µg) and scaffolds without DOX were added to the cells. The DMEM, without the serum, (500 µL/well) was added to the scaffold. A free DOX solution (250 µg/mL) in DMEM was added to the cells as a positive control [19,20]. The cells without samples incubated in DMEM without the serum were used as a negative control. Cells were incubated with scaffold samples for 24 and 48 h at 37 °C, 5% CO_2_. The samples were removed, and cells were washed three times with PBS and were incubated in a culture medium containing 0.5 mg/mL MTT (500 µL/well) for 2 h at 37 °C, 5% CO_2_. After incubation, the MTT solution was removed, and DMSO (350 µL/well) was added to solubilize formazan products. The absorbance was measured at 550 and 650 nm for reference (Spectramax M3, Molecular Devices Inc., San Jose, CA, USA) [19]. The percentage of the cell viability was calculated as a ratio of mean absorbance with respect to the mean absorbance of negative control wells (Equation (5)).

(5) $$Cell\;viability\;\left(\%\right)=\frac{\left(A550-A650control\right)-\left(A550-A650sample\right)}{\left(A550-A650control\right)}\times 100\%$$

### 2.9. Statistical Analysis

A statistical evaluation of data was performed using an analysis of variance (one-way ANOVA). The Newman–Keuls test was used as a post hoc test to assess the significance of differences. In all cases, a value of *p* < 0.05 was accepted as significant.

## 3. Results and Discussion

### 3.1. Hydrogel Scaffold Formation

Polymeric hydrogels can be prepared as three-dimensional hydrophilic networks capable of releasing drugs at the controlled rates. In this study, hydrogel scaffolds were fabricated by modulating the composition of the polymer and the crosslinker to provide properties according to specific applications. The hydrogels were successfully formed from 3% *w*/*v* gelatin, 2% *w*/*v* SCMC, and a 9:1 gelatin/SCMC mixture without glutaraldehyde (Table 1). The hydrogel formation was affected by the addition of glutaraldehyde. The hydrogel did not form when the glutaraldehyde was added to a 2% *w*/*v* SCMC solution, but this effect was not observed when glutaraldehyde was added to a 3% *w*/*v* gelatin solution. The hydrogel formed when a 9:1 and an 8:2 gelatin/SCMC were used to composite the hydrogel in the presence of glutaraldehyde. Increasing the ratio of the SCMC in the gelatin/SCMC polymer solution resulted in an unsuccessful formation of hydrogel in the presence of glutaraldehyde. In the 7:3, 6:4, and 5:5 gelatin/SCMC ratios with the presence of the crosslinker, the hydrogels were not formed. 

Hydrogels can be formed through various methods, including free radical polymerization, irradiation crosslinking, chemical crosslinking and physical crosslinking. The physical crosslinking method involves interactions such as polyelectrolyte complexation (ionic interaction), hydrogen bonding and hydrophobic association. A gelatin-based hydrogel can be prepared through both chemical crosslinking and physical crosslinking techniques. In the case of chemical crosslinking, gelatin-based hydrogel can be developed using dialdehyde (glutaraldehyde) or formaldehyde as a crosslinking agent. With regard to physical crosslinking, a gelatin-based hydrogel can be formed via hydrogen bonding between an electron deficient hydrogen atom and a functional group of high electronegativity [21]. An SCMC is a water soluble polysaccharide which can be fabricated as hydrogel scaffolds [21,22,23]. The hydrogen bonding between polymer chains, i.e., intramolecular hydrogen bonding, of SCMC is hypothesized to be the mechanism of hydrogel formation [24]. With regard to the hydrogel of the gelatin/SCMC mixture formed without using glutaraldehyde, the molecular interaction between the positively charged gelatin and the negatively charged carboxymethyl groups of SCMC have been reported in the preparation of a controlled delivery of microparticles [25]. This might provide one of the mechanisms through which to form hydrogel besides the intramolecular hydrogen bonding formation of each polymer.

Glutaraldehyde can react with various functional groups of proteins, e.g., amine, thiol, phenol and imidazole, since the reactive amino acid sided-chains are nucleophiles [26]. The crosslinking of the gelatin through the addition of glutaraldehyde was largely attributed to the Schiff’s base between the aldehyde and the two free amino groups in lysine or the hydroxylysine of gelatin [27]. SCMC provides some of the hydroxyl groups that react with glutaraldehyde to form hemiacetal or acetal rings [28]. However, the reaction that occurs between the hydroxyl of SCMC and the aldehyde that forms hemiacetals is reversible. The equilibrium may not favor the formation of hemiacetals [29]. In addition, hemiacetals are prone to hydrolysis, leading to an unsuccessful hydrogel formation of SCMC with the addition of glutaraldehyde [28].

**Figure 1:**
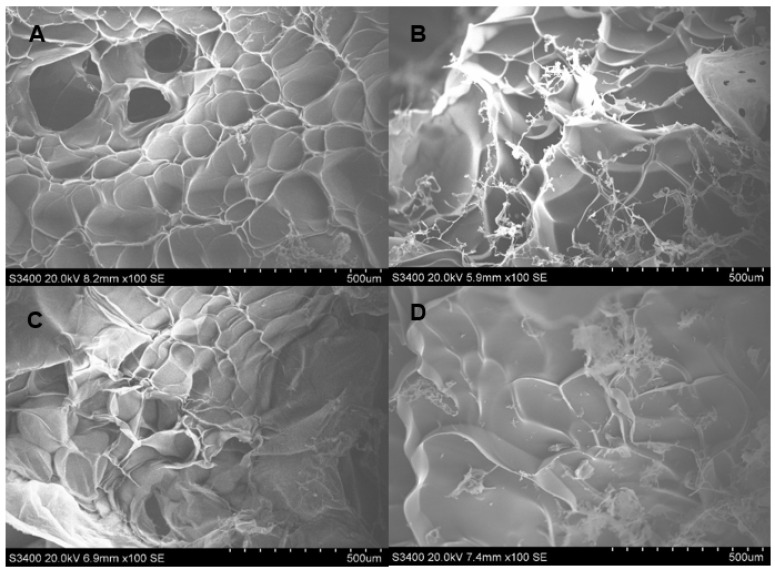
SEM images of hydrogel porous scaffolds made from solution of (**A**) 3% *w*/*w* gelatin (100×); (**B**) 3% *w*/*w* gelatin and 2% glutaraldehyde (100×); (**C**) 3% *w*/*w* gelatin and DOX (100×) and (**D**) 3% *w*/*w* gelatin, DOX, and 2% glutaraldehyde (100×).

**Figure 2:**
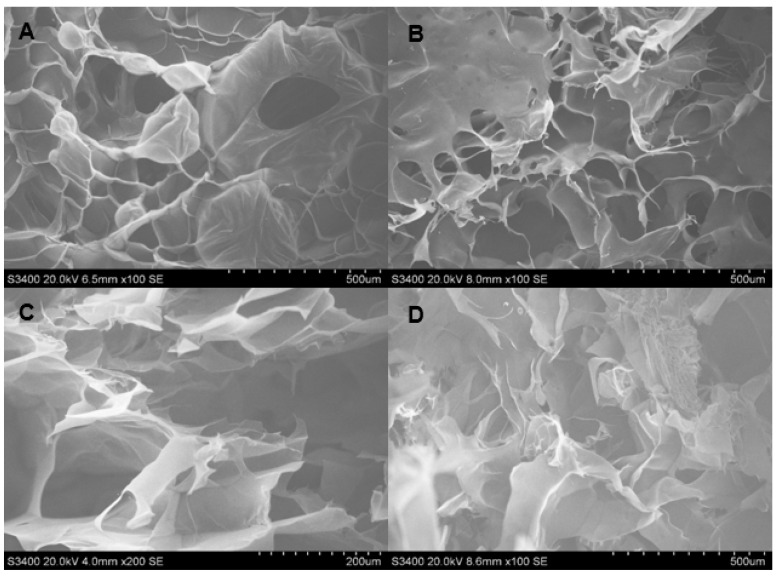
SEM images of hydrogel porous scaffolds made from solution of (**A**) 9:1 gelatin/SCMC solution (100×); (**B**) 9:1 gelatin/SCMC solution and 2% glutaraldehyde (100×); (**C**) 9:1 gelatin/SCMC solution and DOX (100×) and (**D**) 9:1 gelatin/SCMC solution, DOX, and 2% glutaraldehyde (100×).

**Figure 3:**
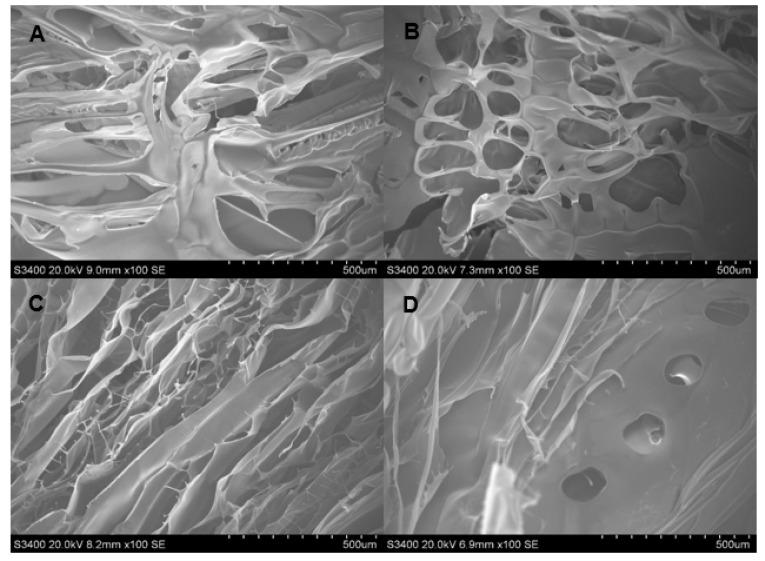
SEM images of hydrogel porous scaffolds made from solution of (**A**) 2% *w*/*v* SCMC solution (100×); (**B**) 2% *w*/*v* SCMC and 2% glutaraldehyde (100×); (**C**) 2% *w*/*v* SCMC and DOX (100×) and (**D**) 2% *w*/*v* SCMC solution, DOX, and 2% glutaraldehyde (100×).

**Figure 4:**
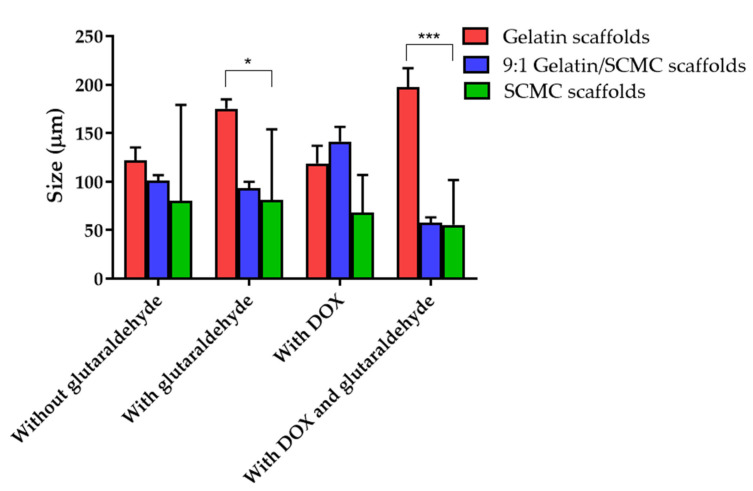
Pore sizes of gelatin, 9:1 gelatin/SCMC, and SCMC scaffolds with and without 2% *w*/*v* glutaraldehyde. Data are expressed as mean ± SEM., * and *** indicate *p* < 0.05 and *p* < 0.001, respectively.

The SEM images showing the morphology and porosity of blank scaffolds and scaffolds containing DOX are presented in Figure 1, Figure 2 and Figure 3. The pore structures of the scaffolds produced by gelatin, SCMC, and the 9:1 ratio gelatin/SCMC mixture, in the presence and absence of glutaraldehyde, are different. The gelatin scaffolds contained regular shapes of pores and uniform pore size (Figure 1). The gelatin/SCMC scaffolds contained a less uniform pore size and shape (Figure 2). The morphology of the pores in the scaffolds prepared using 100% SCMC exhibited an undefined morphology (Figure 3). The average pore size of each type of scaffold is presented in Figure 4. The scaffolds that were made using gelatin possessed the largest pore sizes with the narrowest pore size distribution. The average pore size of scaffolds made from gelatin was in the range of 101–197 µm. The SCMC scaffolds contained the smallest pore size with a wide distribution. The average pore sizes of SCMC scaffolds were in a range of 55–81 µm. The pore sizes of the gelatin/SCMC scaffolds were between those of the gelatin and SCMC scaffolds, at 58–141 µm. All the scaffolds were prepared through the addition of a crosslinker and had some interconnectivity between pores, confirming the use of the crosslinking strategy via glutaraldehyde. However, the morphology and the structure of the scaffolds without the crosslinker addition proved better than those of the scaffolds formed by crosslinking. In addition, to avoid using a chemical crosslinker, all the scaffolds that were used for further investigation were prepared in the absence of glutaraldehyde. The incorporation of DOX into the hydrogel did not affect the pore size and shape of any of the scaffolds.

The pore size and pore porosity depend on the density of the hydrogel and on the presence of a crosslinker. As shown in Figure 4, the pore size of the 9:1 gelatin/SCMC hydrogel scaffold was smaller than that of the gelatin scaffold and larger than that of the SCMC scaffold. However, in the presence of doxorubicin and glutaraldehyde, the pore size was significantly decreased (*p* < 0.0001). These results were consistent with the SEM images presented in Figure 2D. The crosslinker resulted in the formation of an interconnected pore and a smaller pore size [30]. Yang et al. reported that gelatin scaffolds crosslinked with glutaraldehyde had the smallest pore size compared to other crosslinkers [31].

### 3.2. Degree of Hydrogel Scaffold Swelling

**Figure 5:**
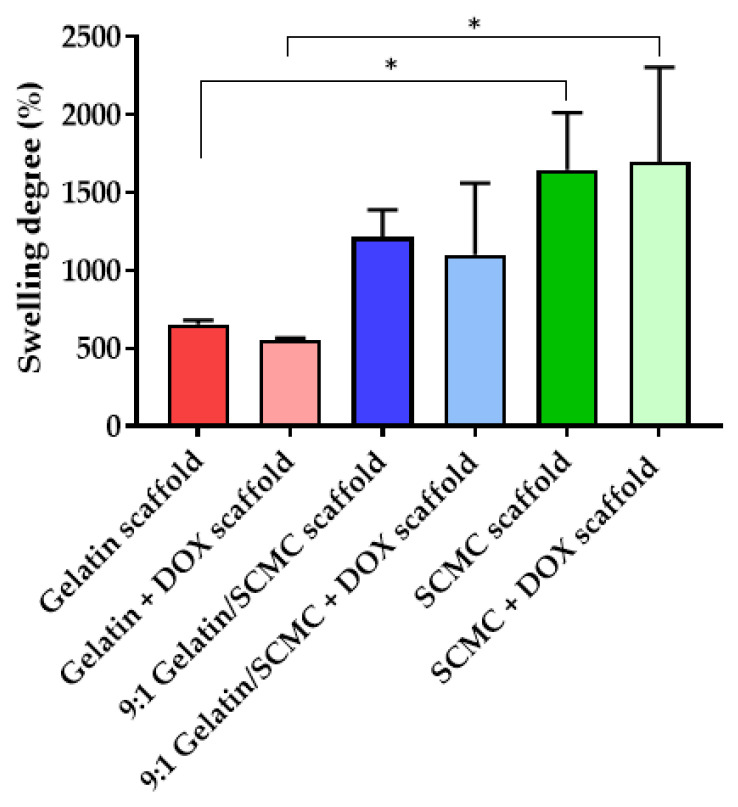
Swelling index (%) of gelatin, 9:1 gelatin/SCMC, and SCMC scaffolds with and without DOX. Data are expressed as mean ± S.D., * indicated *p* < 0.05.

The scaffolds composed of blank gelatin, 9:1 gelatin/SCMC, and SCMC and scaffolds containing DOX were subjected to swelling in PBS, pH 7.4 at 37 °C. Each scaffold showed a different degree of swelling. The results of the swelling study revealed that the SCMC scaffold had the highest swelling degree compared with the 9:1 gelatin/SCMC mixture, and the gelatin scaffolds (Figure 5). The swelling degrees of the gelatin, the 9:1 gelatin/SCMC, and the SCMC were 652.4 ± 26.9%, 1214.0 ± 173.9%, and 1655.8 ± 366.7%, respectively. The addition of DOX to the scaffold did not affect the swelling property of the scaffold. The swelling degree indicated the excellent absorption and retention properties of the scaffolds. The water absorption property depends on the ratio of SCMC in the scaffold. The greater swelling degree of the SCMC scaffolds may be a result of to the higher number of –OH and –COOH groups available for hydrogen bonding with water molecules compared with gelatin [32]. The swelling property of hydrogel is based on water absorption through an open porous structure by the capillary force, the type and the degree of the polymer crosslinking, and the polymer chain length [22,33,34]. DOX is a freely water soluble drug and the amount of DOX added to the scaffold was relatively low compared to the polymer mass. Therefore, the incorporation of DOX in all scaffolds did not influence the swelling properties of all types of scaffolds.

### 3.3. Drug Encapsulation and Loading Efficiency of Hydrogel Scaffolds

**Figure 6:**
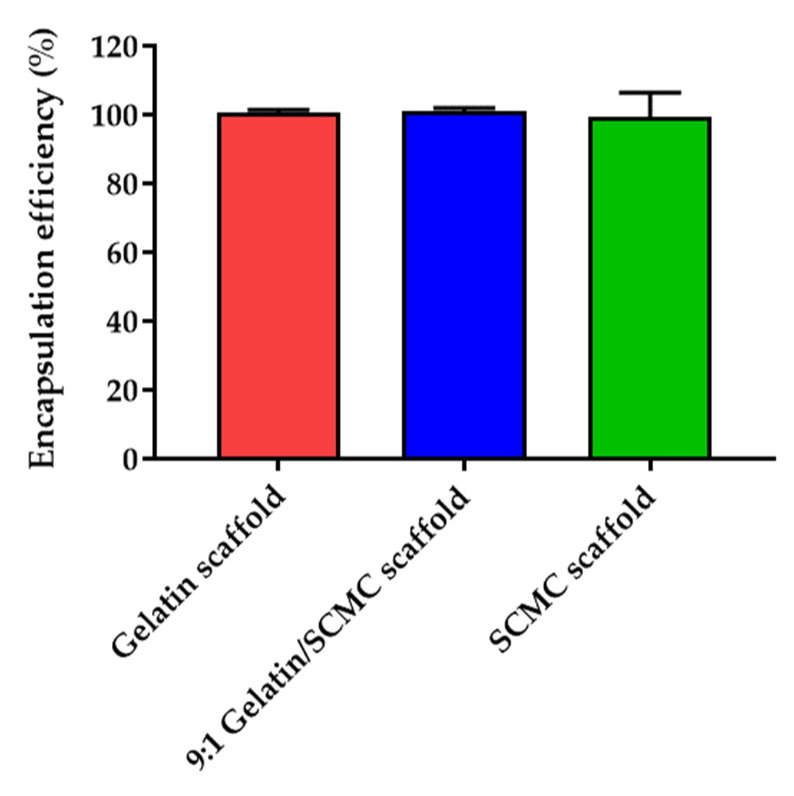
DOX encapsulation efficiency (%) of gelatin, 9:1 gelatin/SCMC, and SCMC scaffolds. Data are expressed as mean ± S.D.

The encapsulation efficiency of the DOX in the gelatin, the 9:1 gelatin/SCMC, and the SCMC scaffolds was 100.7 ± 0.8, 100.9 ± 0.9, and 99.3 ± 7.2% (Figure 6). The amount of DOX embedded in the scaffolds was 167 µg/30 mg of the scaffold, calculated based on the encapsulation efficiency. The high encapsulation efficiency of DOX might be a result of to the incorporation of the DOX, which was performed through the diffusion and absorption processes of the scaffolds. The loading efficiency of the gelatin, the 9:1 gelatin/SCMC, and the SCMC scaffolds were 1.38 ± 0.15%, 0.78 ± 0.07%, and 1.05 ± 0.09%, respectively. 

The drug loading efficiency is usually controlled by the polymer composition and can be estimated relative to the swelling degree in the loading solvent [35]. The drug encapsulation efficiency was around 100% for all of the types of scaffolds, which may be a result of three primary reasons. The first reason is that the amount of DOX loaded into the scaffolds was relatively low. The second reason is that interactions may occur between the DOX and the gelatin or the SCMC, such as an electrostatic interaction, hydrogen bonding, and an intermolecular hydrophobic interaction [36,37,38,39,40]. The third reason is the high swelling degree of the polymer. Both gelatin and SCMC are hydrophilic polymers, therefore, the drug loading capacity in these hydrogels relies on the water solubility of the drug. A DOX is a freely water soluble drug, possessing a water solubility of 50 mg/mL. It was previously shown that drug loading increases relative to the hydrogel swelling [35]. We assumed that the maximum loading efficiency could be higher than the results in this study. The loading of the drug may be designed based on the investigation. In our study, the loading capacity was optimal for the cytotoxicity in the A549 cell line. The drug loading efficiency can be modulated for animal or clinical studies.

### 3.4. In Vitro Drug Release Profiles of Hydrogel Scaffolds

**Figure 7:**
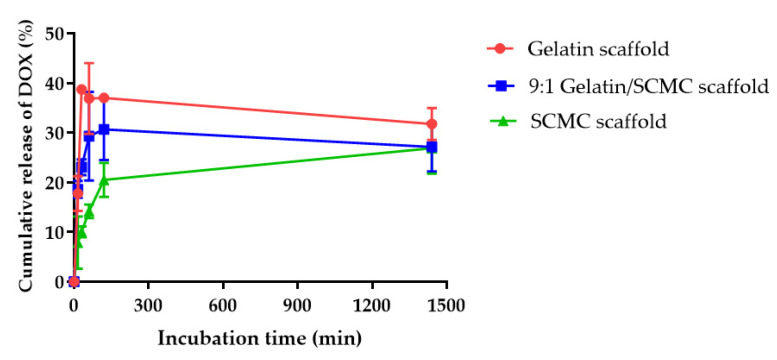
Release profiles of DOX from gelatin, 9:1 gelatin/SCMC, and SCMC scaffolds. Data are expressed as mean ± S.D.

The release profiles of DOX from the gelatin, the 9:1 gelatin/SCMC, and the SCMC scaffolds were investigated at 37 °C for 24 h. Figure 7 shows the comparison of drug release profiles from three different types of scaffolds. The gelatin scaffold displayed the highest rate of drug release followed by the 9:1 gelatin/SCMC scaffold, and the SCMC scaffold, respectively. As shown in Figure 7, the maximum values of the DOX cumulative release from the gelatin, the 9:1 gelatin/SCMC, and the SCMC scaffolds were 31.75 ± 3.24%, 27.16 ± 4.95%, and 26.91 ± 5.15%, respectively. The burst release of the DOX from hydrogel scaffolds was a result of DOX diffusion from the surpassing surface area in the porous scaffolds. The pores of the hydrogel scaffolds increased the potential surface area for drug adsorption. After the hydrogel scaffolds were briefly immersed in PBS, the polymers became hydrated and swelled to form a gel layer on the surface of the system, which could retard the penetration of water into the scaffold core. It was reported that a higher degree of swelling increased the thickness of the gel layer, and reduced the drug release rate [41,42]. The gelatin had a lower swelling degree, compared with the 9:1 gelatin/SCMC, and the SCMC hydrogel scaffolds, resulting in a faster release rate in the first few hours. The incomplete release of drug from the gelatin and the SCMC scaffolds was previously reported [32,43]. The degradation of scaffolds upon biodegradation might induce further release of the drug from the scaffolds [31]. Gelatin scaffolds are susceptible to degradation in the presence of metalloproteinase enzymes within the interstitial space of a tumor [44]. The SCMC scaffolds are biodegradable and can be hydrolyzed through cleaving at the glycosidic linkages [45].

The localized drug delivery system was designed to achieve a high therapeutic concentration of chemotherapeutic drugs in tumor sites and to minimize the systemic side effects of the drug [46]. The chemotherapeutic drugs administered locally usually expose a tumor for a short period of time, leading to an insufficient therapeutic level. The gelatin, the 9:1 gelatin/SCMC, and the SCMC hydrogel scaffolds containing DOX have been developed to provide a biphasic drug release profile which had a burst release of around 37, 30, and 20%, respectively. The gelatin and the SCMC hydrogel scaffolds were previously shown to release higher amounts of the drug due to the biodegradable properties of the polymers, providing a sustained release of the remaining dose over a period of time [46,47]. This could assist in avoiding the repeated administration of the drugs to the patient. Thus, the biphasic drug delivery system, combining an immediate drug release followed by a sustained release, may be favored for local tumor treatment.

The release profile of all the samples suggested a diffusion-controlled model. The release of the drug was usually controlled by the rate of hydrogel scaffold degradation [48]. However, the release profile was not influenced by scaffold degradation during the period of this investigation. In addition to the morphology of the porous scaffold, the degree of swelling may lead to the entrapment of the drug in the hydrogel scaffold. Since the DOX was water-soluble, the increased ability of the scaffold to swell in water resulted in a greater amount of the drug being retained in the hydrogel scaffold. In this study, the amount of DOX loaded into the hydrogel scaffold was relatively low (167 µg/30 mg) to ensure the uniformity of drug distribution throughout the scaffold and to maintain the sink conditions during the release experiment [49]. Therefore, the release profiles of DOX of the three different scaffolds were not different.

### 3.5. Effects of Hydrogel Scaffolds on A549 Cell Viability 

**Figure 8:**
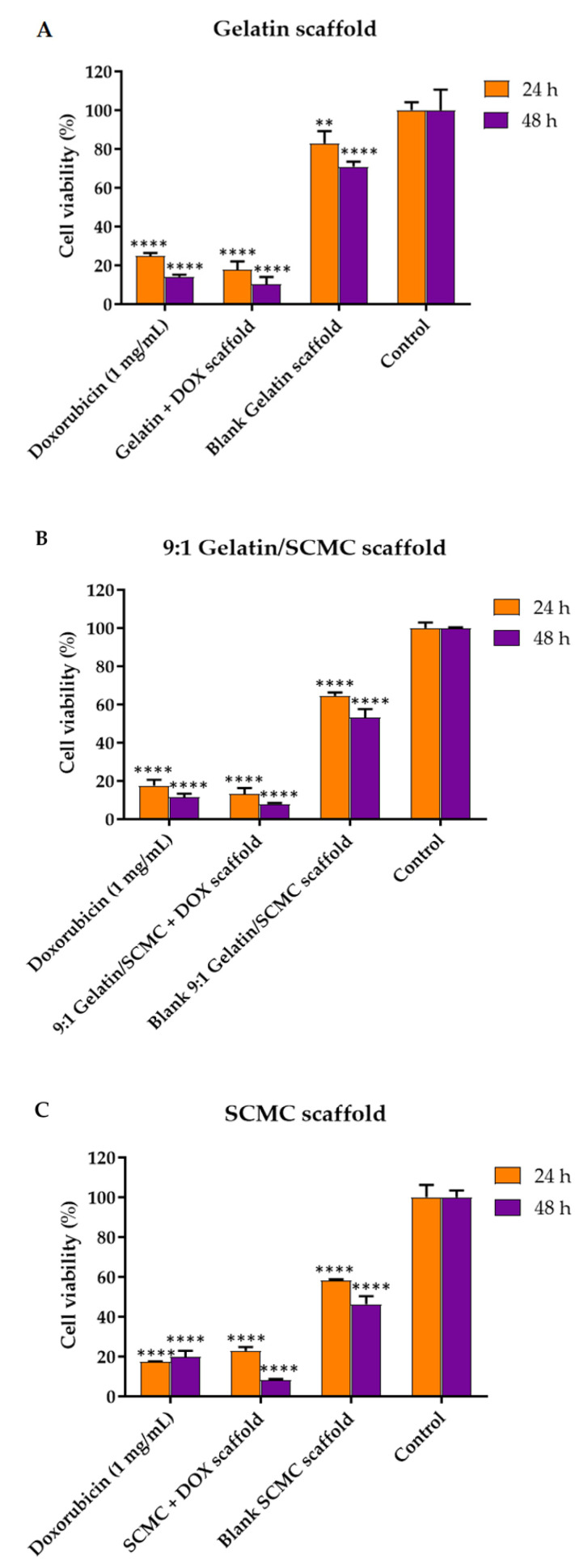
Viability of A549 cells treated with (**A**) gelatin (**B**) 9:1 gelatin/SCMC, and (**C**) SCMC scaffolds containing DOX. Data are expressed as mean ± S.D., ** indicated *p* < 0.01, and **** indicated *p* < 0.0001.

The DOX was incorporated into the porous hydrogel scaffolds to control the drug release to tumors as a localized drug delivery system. Porous hydrogel scaffolds encapsulating the DOX were formed by mixing a 3% *w*/*v* gelatin solution, a 2% *w*/*v* SCMC solution, and a gelatin and SCMC mixture solution at a 9:1 ratio. The DOX exhibited a slow release profile from the scaffold, with around 30% being released within 24 h, indicating the sustained release of DOX to tumors. The A549 lung cancer cell viability, after the application of the scaffold on 24 and 48 h, was analyzed using an MTT assay. The A549 cells significantly decreased in cell number upon exposure to DOX scaffolds (Figure 8). The blank scaffolds prepared from the gelatin, SCMC, and the gelatin/SCMC mixture also reduced cell viability to some extent. Berg et al. reported that the viability of A549 cells exposed to 3D printed alginate/gelatin scaffolds decreased to below 70%, and a substantial loss in their cell number was observed, despite the high content of proteins and growth factors, which was most likely a result of the lower degree of porosity [50]. In our study, A549 cell viability significantly decreased after the treatment with blank gelatin, the gelatin/SCMC mixture, and the SCMC scaffolds from 82%, 85%, to 58%, respectively. This might also result from the smaller porosity and lower degree of porosity observed in the SEM images, resulting in an inadequate supply of nutrients to the cells. The viability of A549 cells, treated with gelatin, gelatin/SCMC, and SCMC scaffolds containing DOX for 24 h were 17, 13, and 22%, respectively. The greater cell viability of A549 after exposure to the SCMC scaffold might result from a lower drug release rate compared with the gelatin and gelatin/SCMC scaffolds. The viability of cells incubated with the DOX solution was comparable to that of cells incubated with the DOX scaffolds. The DOX solution, gelatin scaffold, 9:1 gelatin/SCMC scaffold, the SCMC scaffolds containing DOX, and blank scaffolds decreased A549 cell proliferation in a time-dependent manner. Specifically, the cell viability of A549 cells treated with gelatin, 9:1 gelatin/SCMC, and SCMC scaffolds containing DOX for 48 h was reduced to 1.8, 1.6, and 2.9 fold, respectively, compared with 24-h incubation.

## 4. Conclusions

Porous hydrogel gelatin, SCMC, and gelatin/SCMC mixture scaffolds were developed as a localized drug delivery system for lung cancer treatment. The DOX was loaded within the scaffolds at a high encapsulation efficiency. The ratio of the gelatin and of the SCMC affected the pore size and swelling capacity of the scaffolds. However, it did not affect the drug encapsulation efficiency. All of the scaffolds sustained the release of the drug and effectively killed lung cancer cells. The results suggested these scaffolds to be a promising localized drug delivery system for lung cancer treatment prior to, or after surgery.

## Figures and Tables

**Table 1:** Formation of scaffold prepared from gelatin, sodium carboxymethyl cellulose, and gelatin/SCMC mixture.

Sample Name	3% *w*/*v* Gelatin/2% *w*/*v* SCMC Ratio	Glutaraldehyde 0.2% *w*/*v*	Scaffold Formation
Gelatin	10:0	-	✓
Gelatin + Glutaraldehyde	10:0	✓	✓
SCMC	0:10	-	✓
SCMC + Glutaraldehyde	0:10	✓	✕
Gelatin/SCMC	9:1	-	✓
Gelatin/SCMC + Glutaraldehyde	9:1	✓	✓
Gelatin/SCMC + Glutaraldehyde	8:2	✓	✓
Gelatin/SCMC + Glutaraldehyde	7:3	✓	✕
Gelatin/SCMC + Glutaraldehyde	6:4	✓	✕
Gelatin/SCMC + Glutaraldehyde	5:5	✓	✕

## Data Availability

The data presented in this study are available on request from the corresponding author.
